# Potential preventive and protective perspectives of different spice powders and their mixtures in rat model

**DOI:** 10.1186/s12944-020-01223-9

**Published:** 2020-04-23

**Authors:** Saleeha Hameed, Muhammad Sajid Arshad, Rabia Shabir Ahmad, Ghulam Hussain, Muhammad Imran, Muhammad Umair Arshad, Aftab Ahmed, Muhammad Imran, Ali Imran

**Affiliations:** 1 Institute of Home and Food Sciences, Faculty of Life Science, Government College University, Faisalabad, Pakistan; 2 Department of Physiology, Faculty of Life Sciences, Government College University, Faisalabad, Pakistan; 3 Faculty of Allied Health Sciences, University Institute of Diet and Nutritional Sciences, The University of Lahore-Pakistan, Lahore, Pakistan

**Keywords:** Fenugreek seeds, Black cumin seeds, Efficacy trial, Physical parameters, Lipid profile, Hyperglycemia

## Abstract

**Background:**

The spices based dietary interventions are in lime light among the scientific community owing to their promising therapeutic perspective. The bioactive components in spices can be used to exert various health promoting functions in human body such as prompting weight loss, inhibit diet-induced obesity, hypercholesterolemia, hyperglycemia, allergies and various other maladies. In current study extraction and in vitro characterization of coriander seed (CS), black cumin seed (BCS) and fenugreek seed (FS) polyphenols was conducted for further development of dietary intervention against lipid and glycemia related abnormalities in experimental Sprague Dowley rats fed with control and different spice powder supplemented diets.

**Methods:**

Purposely, extraction of Coriander (CS), Black cumin (BCS) and Fenugreek seeds (FS) were carried out by using water and aqueous methanol (70:30 v/v). Afterwards, the resultant extracts were thoroughly investigated for their antioxidant potential through different indices like TPC, TFC, FRAP and β Carotene Bleaching Assay and ABTS. Furthermore, HPLC quantification were also conducted with special reference to thymoquinone, disogenin, chlorogenic acid, caffeic acid and kaempferol alongside in vitro pancreatic lipase inhibitory activity estimation. Bio-evaluation trial was consisting of three modules i.e. study-I (normal diet), study-II (high cholesterol diet) and study-III (high sucrose diet). Furthermore, rats were sub-divided in five groups in each module on the basis of diet provision including T_0_ (control), T_1_ (Diet containing CS), T_2_ (Diet containing BCS), T_3_ (Diet containing FS) and T_4_ (Diet containing CSP + BCSP + FSP). At the beginning of trial, some rats were dissected to evaluate the baseline values whilst rest of the rats was killed at the termination (56th day). Feed and drink intakes were quantified on daily bases whereas, body weight was calculated weekly. Cholesterol level, serum low density lipoproteins (LDL), high density lipoproteins (HDL), triglycerides, glucose concentration and insulin level of collected sera was measured by standard procedures.

**Results:**

The in vitro characterization showed better extraction of spices antioxidant through aqueous methanol as compared to water. Among the spices, Black cumin seed alone or in combination revealed highest antioxidant activity in T2 (BCS) followed by T4 (CS + BCS), T7 (CS + BCS + FS), T1 (CS), T6 (BCS + FS), T5 (CS + FS) and lowest in T3 (FS). Likewise, the HPLC characterization showed the presence of thymoquinone in BCS, Dosignienin FGS and chlorogenic acid, caffeic acid and kaempferol in the other treatments. Furthermore, all the treatments showed dose dependent inhibition in Pancreatic lipase activity and order of inhibition was BCS > CS + BCS > CS + BCS + FS > CS > BCS + FC > CS + FS > FS. The maximum feed intake, drink intake and weight gain was observed in T0 (control) trailed by T1, T2, T3 and T4 group in experimental study I, II and III, respectively. The resultant diet T4 enhanced the high density lipoprotein from T0 (58.58 ± 2.51) to 61.71 ± 1.62 (T4) in hypercholesterolemia rats whereas in hyperglycaemia rats the HDL was varied from 38.77 ± 1.2 to 40.02 ± 0.99 in T0 and T4, respectively. Similarly, T2 significantly lowered the low density lipoprotein from 62.53 ± 1.22 (T1) & 46.53 ± 0.99 to 54.88 ± 0.52 & 40.94 ± 1.99 (T2) in hypercholesteraemic and diabetic rats. Moreover, T4 treatment showed maximum reduction as 10.01 & 11.53% in respective studies.

**Conclusions:**

The diet prepared from the different combination of spices has been proven effective against Oxidative stress related physiological malfunctioning.

## Background

Plant derived foods with special reference to fruits, vegetables, cereals, spices and herbs hold the therapeutic potential besides the provision of basic nutrition owing to their higher antioxidant capacity. Spices can be used to exert various health promoting functions in human body such as prompting weight loss, inhibit diet-induced obesity, hypercholesterolemia, hyperglycemia, allergies and various other maladies [1]. The antioxidant potential of the spices is due to the presence of many active phytochemicals including alkaloids, vitamins, flavonoids, carotenoids, terpenoids, curcumins, saponin, lignin and plant sterol [2].

Among diverse range of spices, *Nigella sativa* L. (Black cumin seed) is significantly blessed with numerous medicinal and pharmacological activities. The black cumin seeds have been recognized as the source of the active ingredients possessing antioxidant potential [3]. The black cumin seeds contain more than 100 different chemical components, including crude fiber, mucilage, reducing sugars, proteins, resins, flavonoids, alkaloids, sterols, saponins, tannins, organic acids alongside high content of unsaturated fatty acids. Other bioactive ingredients include linoleic acid, oleic acid, calcium, potassium, iron, zinc, magnesium, selenium, vitamin A, vitamin B_2_, niacin and vitamin C [4, 5]. The black seeds oil contains compounds like thymoquinone (30–48%), carvacrol (6–12%), p-cymene (7–15%), t-anethole (1–4%), 4-terpineol (2–7%) and sesquiterpene longifolene (1–8%) [6]. However, thymoquinone and its derivatives thymohydroquinone, dithymoquinone and thymol are the most putative pharmacologically active constituents of BCS [7]. Likewise, *Trigonella foenum graecum* (Fenugreek seed) belongs to family Fabaceae (Leguminosae) is an annual herbaceous plant, widely grown in Asia, has known to its therapeutic potential owing to its rich antioxidant profile deemed with saponins, coumarin, fenugreekine, nicotinic acid and phytic acid [8]. These arrays of compounds assist in lowering blood LDL-cholesterol levels, inhibit re-absorption of bile in the colon and bind the toxins in food which may reliefs’ colon mucusa from cancers [9]. *Coriandrum sativum* L. (Coriander seeds) includes flavonoids (quercetin and isoquercetin), polyphenols (rutin, caffeic acid derivatives, ferrulic acid, gallic acid and chlorogenic acid), β- caroteinoids and tannins. Coriander seeds are also rich in monounsaturated fatty acids (78.2%), saturated fatty acids (6.55%) and polyunsaturated fatty acids (15.08%) [10].

Considering the composition of the spices and presences of appreciable amount of nutraceutical components, the present study was conducted to explicate the in vitro characterization and hypolipidemic and hypoglycemic potential of coriander seed powder, black cumin seed powder and fenugreek seed powder in experimental Sprague Dowley rats fed with control and different spice powder supplemented diets.

## Methods

Purposely, three types of spices were analyzed to determine the protective role of spices against hypercholesterolemic and hyperglycemic rats. Seeds of three different spices i.e. coriander; black cumin and fenugreek were procured from Vegetable Research Section, Ayub Agriculture Research Institute (AARI), Faisalabad, Punjab, Pakistan. Various analytical and HPLC grade reagents and standards were purchased from Merck (Merck KGaA, Darmstadt, Germany) and Sigma-Aldrich (Sigma-Aldrich Tokyo, Japan). For bio-evaluation study, Sprague Dawley rats were housed in the Animal Room of College of Pharmacy, Government College University Faisalabad, Pakistan. For efficacy trial, diagnostic kits were purchased from Sigma-Aldrich, Bioassay (Bioassays Chemical Co. Germany) and Cayman Chemicals (Europe).

### Raw materials processing

The spices were washed thoroughly under running tap water to remove adhered dirt, dust and other foreign debris. After washing, the seeds were dried at room temperature for few days. The dried materials were ground further to fine powder by using a small laboratory grinder (Panasonic, Japan, Model MJ-W176P) and passed through a sieve for further refining. After preparation of powder for each category, it was packed separately in air-tight plastic jars for further analysis.

### In vitro characterization of spices extracts

In this segment the spices were subjected for their polyphenol extraction by adapting the solvent extraction method by utilizing water and aqueous methanol as extraction medium. Afterwards, the resultant extracts were probed for their lipid peroxidation diminishing indicators through total Phenolic estimation, total antioxidant activity, ferric reducing antioxidant power and ABTS assay determination as described below:

### Determination of Total Phenolics (TP)

Total polyphenols (TP) were measured by using Folin-Ciocalteu method following the protocol of Singleton [11] and absorbance was recorded at 765 nm with UV/Visible Spectrophotometer (IRMECO, U2020) against control. Total polyphenol was estimated as gallic acid equivalent (mg gallic acid/100 g).

### Determination of Total flavonoid (TF)

The total flavonoid content was determined according to the colorimetric method [12]. The absorbance was determined at wavelength 510 nm. The TFC was expressed in quercetin equivalents per gram (mg quercetin/100 g).

### Carotene bleaching assay

Antioxidant activity of spice extracts was estimated based on coupled oxidations of β- carotene and linoleic acid [13]. Briefly, β-carotene 2 mg was dissolved in 20 mL chloroform, 40 mg linoleic acid and 400 mg Tween20. After removing chloroform, 3 mL of the prepared emulsion was added in 0.10 mL sample and placed in a water bath for 120 min. Oxidation of ß-carotene was determined spectrophotometricaly at 470 nm.

### Ferric reducing antioxidant power (FRAP)

The ferric reducing power of extracts was estimated according to the protocol of [14]. The absorbance was measured at 700 nm. During the analysis, an increase in the absorbance (A) of the reaction mixture indicated the reducing power.

### ABTS (2,2′-azino-bis, 3-ethylbenzothiazoline-6-sulfonic acid) assay

An ABTS assay was measured following the protocol of Böhm [15]. The absorbance was determined at 734 nm using Spectrophotometer (CECIL CE7200).

### HPLC quantification of bioactive compounds

Different preparation of spice extracts was subjected to HPLC quantification to estimate the comparative abundance of bioactive molecules. Identification of flavonol (kaempferol) and phenolic acid (chlorogenic acid & caffeic acid) were carried out by adapting the guidelines of [16]. The Conditions for HPLC were HPLC (PerkinElmer, Series 200, USA) was comprised of SCL- 10A system control unit, UV-visible detector (SPD- 10AUV λ max 360 nm), Rheodyne injector, CTO-10A column oven and LC-10 AS pumps. Moreover, Thymoquinone quantification was performed using the method of Ghosheh [17]. While, Disogenin in fenugreek seed were estimated by using the guidelines of [18].

### In vitro pancreatic lipase inhibitory activity

To evaluate the in vitro antihyperlipidemic effect of Spices, the pancreatic lipase inhibitory activity was monitored by adapting the guidelines of Kubdi et al. [19].

### Bio-evaluation studies

The study program was designed after the review and approval of ethical guidelines set by parent institute which are in compliances with international standards (ERC 1009). For bio-evaluation, seed powder of spices was probed for their anti-diabetic and anti-hyperlipidemic potential in the 8 weeks study on rat feeding trial. Albino rats (150) were procured from National Institute of Health, Islamabad and housed in the Animal Room of College of Pharmacy at Government College University, Faisalabad. Initially, the rats were acclimatized by feeding basal diet for one week period. During the experiment, the environmental conditions were maintained i.e. temperature 23 ± 2 °C and relative humidity 55 ± 5% with 12 h light-dark period. At the commencement of trial, some rats (total 15 rats and average of results were considered as base line trend) were sacrificed to establish the baseline trend. For the induction of hypercholesterolemia initially high Cholesterol @ 1.5% and cholic acid @ 0.5% were administrated for a period of 14 days. During that tenure Cholesterol and LDL level were observed to estimate the onset of Hypercholesterolemia. Likewise, High sucrose diet was used as an agent to induce hyperglycaemia @40% for the similar tenure and observed the values for Glucose. Afterwards when values of mentioned tests deviate 50% from normal then the original study was started. In the animal modelling, five groups of rats were formed in three different studies assigning 10 rats (Sample size according to power analysis) in each group (Table 1) to determine effect of spices against selected maladies. For control group, experimental diet was prepared by using corn oil (10%), corn starch (66%), protein (10%), cellulose (10%), mineral (3%) and vitamin mixture (1%). Whereas, for T_1_, T_2_, T_3_, T_4_, contains coriander seed powder (CSP), black cumin seed powder (BCSP), fenugreek seed powder (FSP) and mixture of all three spices @ 1 g/Kg B. W, respectively (Table 2). At the termination of the study, overnight fasted rats were decapitated and blood was collected. For serum collection, blood samples were subjected to centrifugation using centrifuge machine @ 4000 rpm for 6 min. The respective sera samples were examined for various biochemical assays by using Microlab 300, Merck, Germany. Different biochemical parameters including lipid profile alongside glucose & insulin, serum urea and creatinine status, liver function test, and hematological analysis were accessed using respective commercial kits.

**Table 1:** Diet and treatment plan used for bio-efficacy trial

Diets	Study-I (Normal experimental rats fed on Normal diet)					Study-II (Hypercholesterolemic rats fed on high cholesterol diet)					Study-III Diabetic rat fed on High Sucrose diet)				
Groups	1	2	3	4	5	1	2	3	4	5	1	2	3	4	5
Drinks	T_0_	T_1_	T_2_	T_3_	T_4_	T_0_	T_1_	T_2_	T_3_	T_4_	T_0_	T_1_	T_2_	T_3_	T_4_

Each group consist of 10 Sprague dawley rats in each. All the studies were independent having their own control and in all studies all the animal were provided respective treatments for a period of 56 days

T_0_: Control (Group rely on respective Experimental Diet+ without active ingredient) theaflavins supplementation @ 1 g

T_1_: Group rely on respective Experimental Diet+ Coriander seed supplementation @ 1 g/Kg B.W

T_2:_ Group rely on respective Experimental Diet+ Black Cumin seed supplementation @ 1 g/Kg B.W

T_3_: Group rely on respective Experimental Diet+ Fenugreek seed supplementation @ 1 g/Kg B.W

T_4_: Group rely on respective Experimental Diet+ Coriander Seed+ Black cumin seed + Fenugreek seed supplementation @ 0.333 + 0.333 + 0.333 g/Kg B.W

**Table 2:** Mean values for HPLC quantification of Chlorogenic acid, Caffeic acid, Kaempferol, Thymoquinone and Disogenin (μg/g) in different spices

Treatments	Chlorogenic Acid	Caffeic Acid	Kaempferol	Thymoquinone	Disogenin
T_1_	139.6 ± 7.72a	83.73 ± 2.39a	228.6 ± 9.72a	N.D	N.D
T_2_	101.5 ± 5.43c	4.09 ± 0.09e	6.02 ± 0.03e	5.13 ± 0.06a	N.D
T_3_	40.3 ± 0.79 g	3.06 ± 0.06 g	5.59 ± 0.02 g	N.D	27.72 ± 0.52a
T_4_	109.8 ± 6.52b	22.83 ± 2.37c	114.6 ± 4.84b	2.49 ± 0.03c	N.D
T_5_	88.6 ± 2.26e	23.22 ± 2.53b	112.1 ± 4.62c	N.D	11.27 ± 0.35c
T_6_	68.8 ± 1.59f	3.46 ± 1.45f	5.82 ± 0.06f	2.54 ± 0.02b	12.31 ± 0.37b
T_7_	93.4 ± 2.96d	30.05 ± 1.36d	81.5 ± 1.39d	1.69 ± 0.01d	8.39 ± 0.15d

One way anova was applied to check the overall behavior of the study parameter to elaborate the effect of treatments on HPLC quantification. To evaluate the differences among the mean LSD test was applied. Values in same column within each parameter with different letters were significantly different from each other (*p* ≤ 0. 05)

T_1_ = Coriander Seed (CS) extract

T_2_ = Black Cumin Seed (BCS) extract

T_3_ = Fenugreek Seed (FS) extract

T_4_ = CS + BCS extract

T_5_ = CS + FS extract

T_6_ = BCS + FS extract

T_7_ = CS + BCS + FS extract

### Study I: rats fed with normal diet

Initially, efficacy trial was conducted in normal rats given normal diet. The composition of normal diet was corn oil (10%), corn starch (66%), protein (10%), cellulose (10%), mineral (3%) and vitamin mixture (1%).

### Study II: hyperlipidemic rats

In study II, high fat diet containing 1.5% cholesterol and 0.5% Cholic acid was given to rats to raise their lipid profile and the effect of spices was noted on the induced trait.

### Study III: hyperglycemic rats

In study III, high sucrose diet containing 40% sucrose was given to the normal rats to induce diabetes and determine its effect on serum glucose and insulin level. At the same time, effect of spices on the induced trait in respective groups of rats was assessed.

### Feed and water intake

The experimental rats were monitored for the feed and water intake throughout the trial. The gross feed intake of each group was calculated every day, excluding the spilled diet throughout the study period. The net water intake was also recorded on daily basis by measuring the difference in graduated bottles.

### Body weight gain

The gain in body weight for each group of rats was monitored on weekly basis to estimate any suppressing effect of spices formulations.

### Serum lipid profile

Cholesterol level of collected sera was measured by liquid cholesterol CHOD–PAP method according to the guidelines of Kim [20]. Serum low density lipoproteins (LDL) were estimated following the protocol of Kim [20]. Accordingly, the high density lipoproteins (HDL) were assessed by Cholesterol Precipitant method [21]. The triglycerides in the collected sera were measured by liquid triglycerides (GPO–PAP) method as described by [20].

### Glycemic indicators

In each group, glucose concentration was estimated by GOD-PAP method as described by Katz [22], whereas, insulin level was estimated by following the instructions of Ahn [13].

### Liver functioning tests

For liver soundness, alanine transferase (ALT), aspartate transferase (AST) and alkaline phosphatase (ALP) were estimated [23]. The ALT and AST levels were measured by dinitrophenylhydrazene (DNPH) through Sigma Kits 58–50 and 59–50, respectively whereas; Alkaline Phosphatas-DGKC was used for ALP assessment.

### Total antioxidant capacity (TAC)

The Total Antioxidant activity was measured by using the Trolox equivalent antioxidant capacity or TEAC and Glutathione estimation [24].

### The TEAC assay

Briefly, The TEAC assay is commonly based on the principle that when 2, 2′-azinobis 3 ethylbenzothiazoline-6-sulfonate (ABTS) is incubated with H_2_O_2_, a radical of ABTS (ABTS•+) is formed. The ABTS• + is blue-green in color and has the maximum absorption at 650, 734, and 820 nm. The antioxidants present in the sample decrease the ABTS• + and suppress the color production which is inversely proportional to the total antioxidant capacity of serum sample. The rate of reaction is generally calibrated with the Trolox which is a water-soluble equivalent of vitamin-E, and the results are measured as mmol Trolox equivalent/L [25].

### Glutathione contents

Glutathione contents were assessed by adapting the guidelines as mentioned by [26]. The reaction of product of GSH + DTNB in the protein free supernatant was estimated at 412 nm and expressed as nmol/mg protein.

### Haematological aspects

Red blood cells indices including total red blood cells (TRBCs), hemoglobin (hb), hematocrit (Hct) and mean corpuscular volume (MCV) were estimated. Likewise, white blood cell indices including monocytes, lymphocytes and neutrophils were measured by using Automatic Blood Analyzer (Nihon Kohden, Japan). Indicators of electrolytes balance like Na, K and Ca of collected blood samples were also probed by their respective methods [23, 27].

### Statistical analysis

The data regarding different treatments was obtained by applying completely randomized design (CRD) and further subjected to statistical analysis using Statistical Package (Microsoft Excel 2016 and Statistix 9.1). Level of significance was determined (ANOVA, LSD for comparison) using 2-factor factorial CRD where applicable following the principles outlined by Steel [28].

## Results

### In vitro antioxidant profiling

**Fig. 1:**
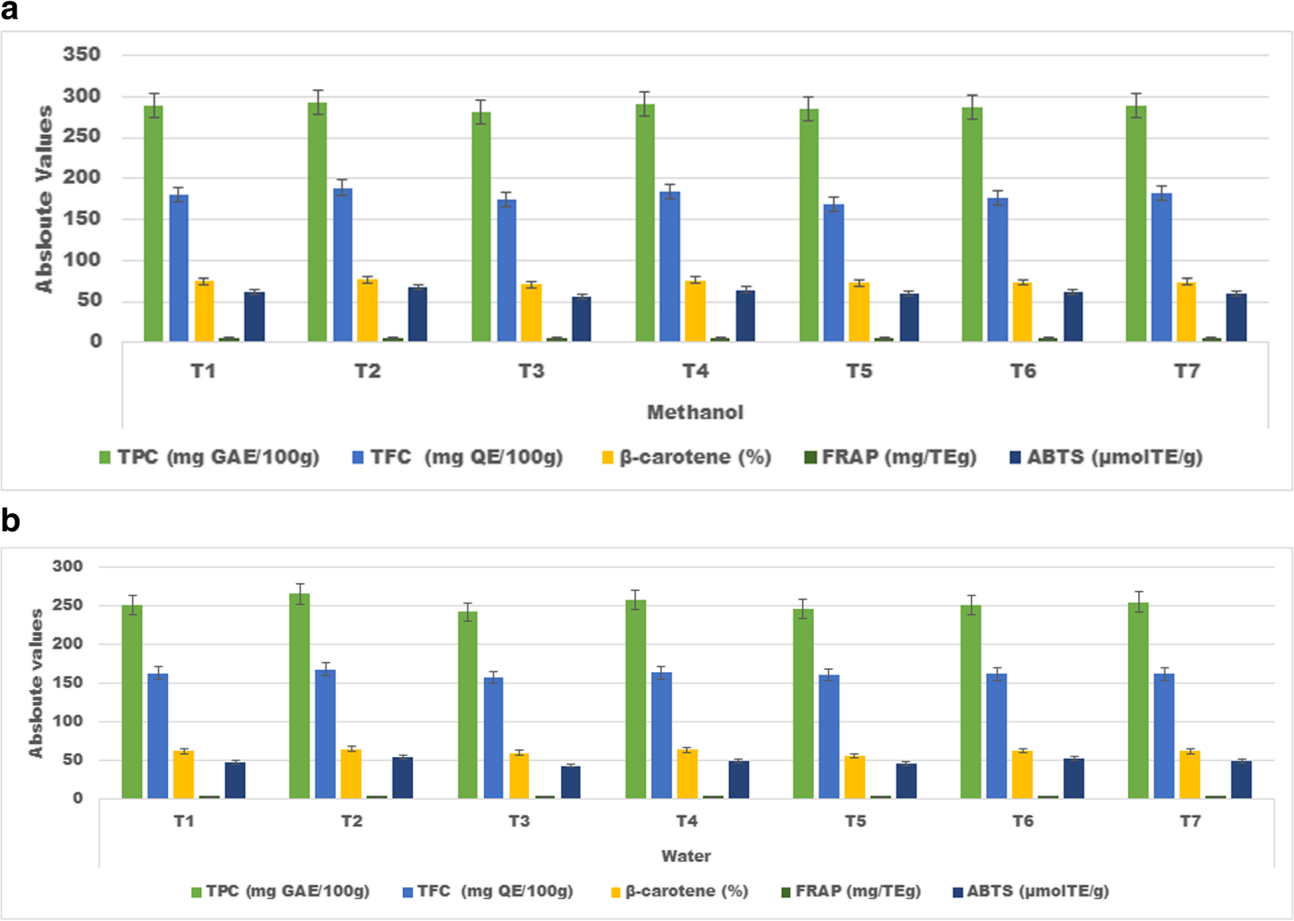
**a**, **b**: Absolute values for different antioxidant indices carried out of water and methanolic extracts of different spices in alone and in combination. Two way ANOVA was applied to check the overall behaviour of the study parameter to elaborate the effect of treatments and solvent on different antioxidant indices . To evaluate the differences among the mean LSD test was (*p* ≤ 0. 05)

Antioxidant activity estimation is an indication of the tested compound effectiveness to halt the oxidation process which can further validate in a biological system. In current research different antioxidant indices were applied to evaluate the effectiveness of tested compounds against lipid peroxidation. it is deduced from the Fig. 1a and b that both treatments (*p* ≤ 0. 001) and solvents (*p* ≤ 0. 003) had significant impact. Amongst the solvents the methanolic extract (70:30 v/v) exhibited more pronounced impact for polyphenol extraction and antioxidant estimation as compared to water. Whereas, in treatments the order of effectiveness for TPC, TFC, FRAP, β Carotene Bleaching Assay and ABTS was BCS > CS + BCS > CS + BCS + FS > CS > BCS + FC > CS + FS > FS. It is further evident that the combination of some spices elucidated better results as compared to alone thus showing synergism.

### HPLC quantification of bioactive phytochemicals

**Fig. 2:**
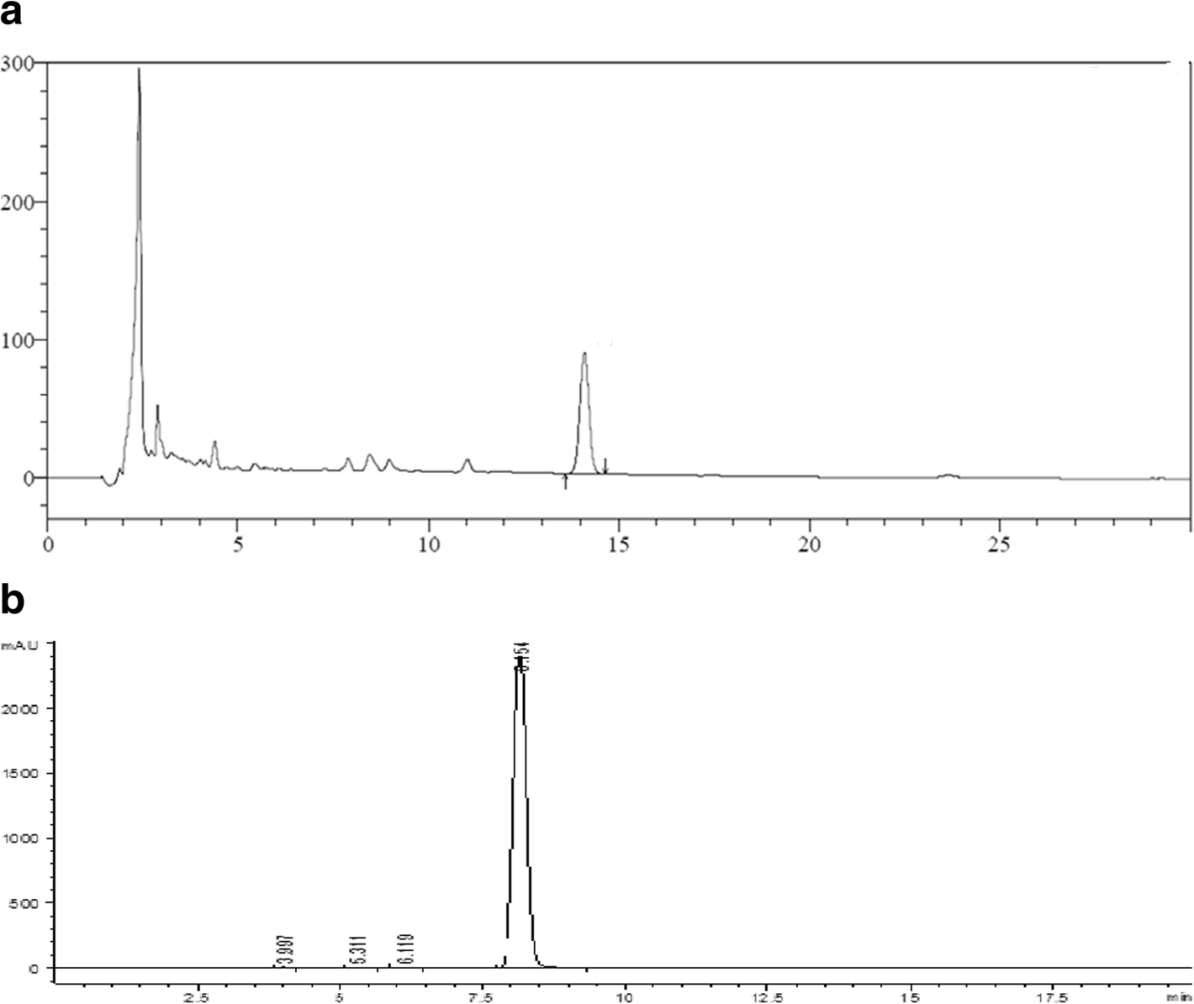
**a**, **b:** HPLC chromatograms of Disogenin and Thymoquinone

HPLC quantification of different spices extracts for their bioactive constituents like Chlorogenic Acid, Caffeic Acid, Kaempferol, Thymoquinone & Disogenin were carried. The results (Methanolic extracts) showed the presence of Chlorogenic Acid, Caffeic Acid, Kaempferol in all treatments however, higher amount was observed in CS extract. In contrary, Thymoqunine was only observed in BCS and their combination. Likewise, Diosgenin was detected in FS and its combination (Table 2) Fig. 2.

### In vitro pancreatic lipase (PL) inhibitory activity

**Fig. 3:**
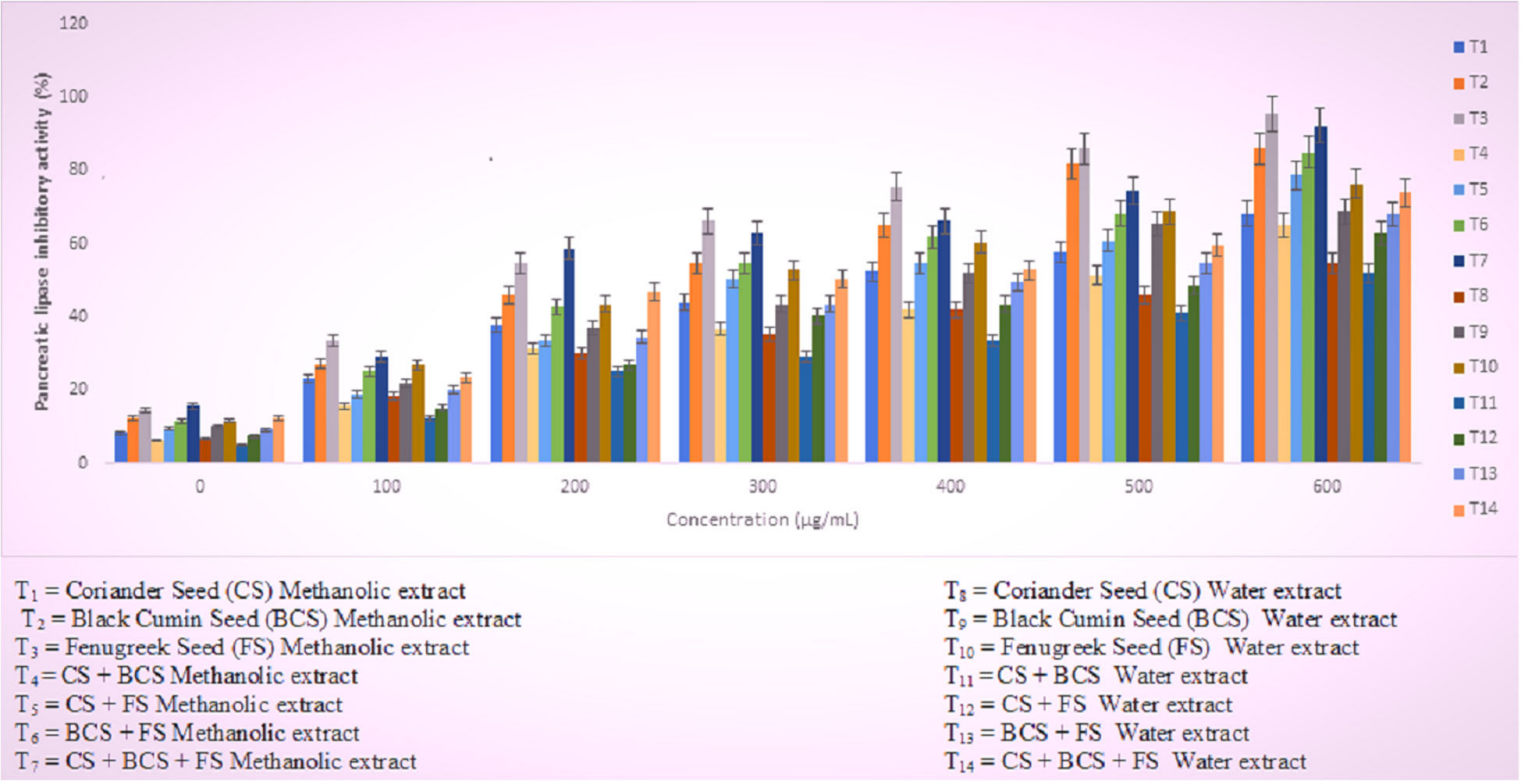
Pancreatic lipase inhibitory activity (%) of methanolic and water extracts of spices alone or in combination was evaluated on dose dependent manner by varying the concentration from 0 to 600 microgram/mL. Values are mean ± SEM (*n* = 03)

To elucidate the mechanistic phenomenon associated with hypolipidemic perspective of spices, in vitro pancreatic lipase inhibitory activity was performed. From Fig. 3 it was evident that all the treatments imparted significant (*p* ≥ 0.002) dose dependent inhibition in pancreatic lipase activity. It is also well reflected that the methanolic extract performed better as compared to water extracts. The order of inhibition was BCS > CS + BCS > CS + BCS + FS > CS > BCS + FC > CS + FS > FS.

### In vivo therapeutic potential estimation

#### Effect of treatments on feed intake, drink intake and body weight

**Fig. 4:**
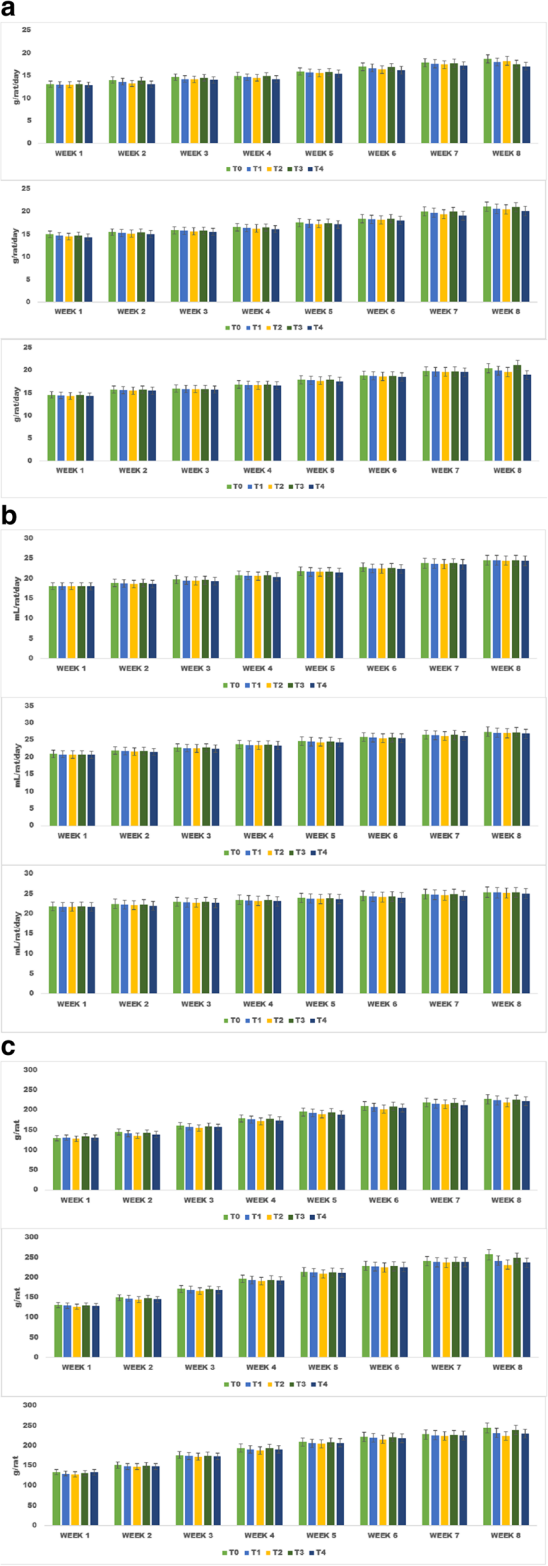
**a** Feed intake by the experimental rats (g/rat/day) (A: fed with normal diet; B: fed with high cholesterol diet; C: fed with high sucrose diet). **b** Water intake by the experimental rats (mL/rat/day) (A: fed with normal diet; B: fed with high cholesterol diet; C: fed with high sucrose diet). **c** Weight gain by the experimental rats (g/rat) (A: fed with normal diet; B: fed with high cholesterol diet; C: fed with high sucrose diet)

The mean values elucidated a significant (*p* ≥ 0.004) reduction on feed intake of rats in all studies owing to the consumption of spices based dietary intervention as compared to control. The highest feed intake was observed in T_0_ (control) followed by T_3_ (Containing Fenugreek seeds), T_1_ (Cumin Seed), T_2_ (Black cumin seed) and least feed intake was recorded in group fed on combination of spices T_4_(CS + BCS + FS). Likewise pattern was observed for drink intake, highest in control as compared to experimental diets in all studies and similar order for reduction was recorded. However, in all studies the feed and drink intake were gradually increased with the passage of time among the studied groups however, the enhancement was more pronounced in T_0_ (control) then the rest (Fig. 4a and b).

Body weight of rats in all studies showed significant (*p* ≥ 0.001) differences due to the treatments. The weight gain increases significantly with passage of time however, maximum increase was observed in control diet (T_0_) feed group followed by T_3_, T_1,_ T_2_ & T_4._ The mean values of body weight in different rat groups T_0_, T_1_, T_2_, T_3_ and T_4_ were 130 ± 6.32, 132 ± 7.22, 129 ± 5.85, 134 ± 8.74 and 131 ± 4.85 g/rat, respectively that subsequently increased to 227 ± 10.41 g/rat (T_0_), 224 ± 11.02 g/rat (T_1_), 219 ± 9.62 g/rat (T_2_), 226 ± 8.72 g/rat (T_3_) and 222 ± 15.21 g/rat (T_4_) at the termination of study (Study I). Rats fed on high cholesterol diet (study II) showed the highest weight gain in T_0_ (131 ± 8.08 & 257 ± 7.56 g/rat) at 8th week followed by T_2_ (127 ± 8.06 & 232 ± 9.99 g/rat), T_4_ (128 ± 7.85 & 237 ± 7.89 g/rat), T_1_ (129 ± 6.85 & 242 ± 7.89 g/rat) and T_3_ (130 ± 7.79 & 249 ± 9.89 g/rat). Likewise, in study III (high sucrose diet), T_0_ exhibited maximum weight gain (134 ± 0.03 g/rat) followed by T_1_ (130 ± 7.22 g/rat), T_2_ (128 ± 8.52 g/rat), T_3_ (131 ± 8.75 g/rat) and T_4_ (133 ± 9.55 g/rat) (Fig. 4c).

#### Effect of treatments on lipid profile

The Mean values (Table 3) expounded significant impact of treatments on lipid profile of rats in all studies. However, the more potent effect was observed in study II (Hypercholestrolemic rats) followed by study III (Hyperglycaemic rats) & study I (normal rats). Likewise among the treatments, T_2_ (BCS supplemented diet) showed highest impact on lipid profile followed by T4 (BCS + CS + FGS supplemented diet), T_1_ (CS supplemented diet) & T_3_ (FGS supplemented diet).

**Table 3:** Effect of spices supplemented diet on serum triglycerides (TG), low density lipoprotein (LDL-C), high density lipoprotein (HDL-C), total cholesterol (TC), glucose, insulin, serum liver enzyme and antioxidant status in normal, hypercholestrolemic and hyperglycemic rats at the termination of the study

**Cholesterol mg/dL)**	77.80 ± 3.56	T0	81 ± 4.5	155 ± 11.2a	101 ± 7.1a
		T1	79.37 ± 4.3	142.37 ± 9.5bc	93.92 ± 5.2bc
		T2	78.21 ± 4.2	136.48 ± 6.5d	90.89 ± 4.2d
		T3	79.80 ± 3.3	145.20 ± 6.2b	95.82 ± 3.2b
		T4	78.56 ± 3.2	138.80 ± 7.5 cd	92.50 ± 3.9 cd
**HDL (mg/dL)**	32.56 ± 3.56	T0	39.85 ± 1.25	59.23 ± 2.51b	38.77 ± 1.23b
		T1	39.98 ± 1.12	61.65 ± 3.22a	39.94 ± 1.56a
		T2	40.24 ± 1.62	61.87 ± 2.61a	40.15 ± 1.99a
		T3	39.93 ± 0.05	61.59 ± 1.92a	39.91 ± 0.07a
		T4	40.08 ± 1.05	61.71 ± 1.62a	40.02 ± 0.99a
**LDL-C (mg/dL)**	26.56 ± 3.56	T0	28.09 ± 0.56	62.53 ± 1.22^a^	46.53 ± 0.99a
		T1	27.11 ± 0.98	57.81 ± 1.01^b^	41.87 ± 1.55c
		T2	26.63 ± 0.12	54.88 ± 0.52^c^	40.94 ± 1.99c
		T3	27.33 ± 0.23	60.50 ± 2.25^a^	43.03 ± 1.23b
		T4	26.83 ± 0.55	55.94 ± 0.09^c^	41.41 ± 1.22c
**Triglyceride(mg/dL)**	58.26 ± 3.56	T0	67.37 ± 3.23	99.23 ± 4.52^a^	77.46 ± 2.92a
		T1	66.01 ± 2.52	92.76 ± 3.63^b^	74.09 ± 3.35b
		T2	65.05 ± 2.25	91.75 ± 2.26^b^	73.34 ± 1.77b
		T3	66.38 ± 1.55	92.96 ± 4.55^b^	74.86 ± 3.36ab
		T4	65.34 ± 2.22	92.27 ± 4.29^b^	73.58 ± 3.79b
**Glucose (mg/dL)**	79.63 ± 3.72	T0 (control)	86.53 ± 3.32	101.23 ± 4.55a	137.53 ± 6.52a
		T1	84.79 ± 4.12	94.13 ± 3.45bc	126.32 ± 5.45b
		T2	83.93 ± 2.29	92.72 ± 3.99 cd	125.14 ± 4.55bc
		T3	85.25 ± 1.22	96.05 ± 3.69b	127.47 ± 5.21b
		T4	83.54 ± 1.32	91.09 ± 4.5d	122.97 ± 6.01c
**Insulin (μU/Ml)**	6.23 ± 0.56	T0 (control)	8.23 ± 0.5	8.95 ± 0.3a	12.56 ± 0.7b
		T1	8.26 ± 0.4	9.06 ± 0.5a	12.94 ± 0.9a
		T2	8.28 ± 0.4	9.15 ± 0.4a	12.97 ± 0.7a
		T3	8.25 ± 0.1	9.04 ± 0.4a	12.93 ± 0.5a
		T4	8.31 ± 0.2	9.23 ± 0.3a	13.09 ± 0.6a
**AST(IU/L)**	101.25 ± 2.36	T0 (control)	108.51 ± 4.36	138.56 ± 6.29a	119.47 ± 4.99a
		T1	106.51 ± 4.36	133.07 ± 5.69ab	114.43 ± 5.56b
		T2	105.85 ± 4.26	132.24 ± 5.57	113.54 ± 5.41b
		T3	106.94 ± 4.22	133.72 ± 5.49ab	114.82 ± 5.62ab
		T4	105.04 ± 4.29	130.89 ± 6.99b	112.79 ± 5.81b
**ALT(IU/L)**	40.12 ± 1.01	T0 (control)	51.52 ± 2.29	59.38 ± 1.29a	47.26 ± 5.22a
		T1	50.23 ± 3.26	56.42 ± 3.39b	44.89 ± 5.56b
		T2	49.97 ± 3.26	56.27 ± 1.57b	44.66 ± 5.41b
		T3	50.48 ± 4.22	56.58 ± 4.49b	45.00 ± 5.62b
		T4	49.86 ± 2.29	56.06 ± 3.99b	44.52 ± 6.01b
**ALP(IU/L)**	130.24 ± 2.01	T0 (control)	156.52 ± 5.22	237.14 ± 9.27a	223.42 ± 9.56a
		T1	152.48 ± 4.79	208.70 ± 8.23bc	192.16 ± 7.12bc
		T2	149.49 ± 4.21	206.26 ± 8.01c	188.88 ± 6.36 cd
		T3	153.42 ± 4.12	213.38 ± 8.56b	196.86 ± 6.99b
		T4	147.09 ± 3.99	197.96 ± 6.69d	184.23 ± 6.01d
**Glutathione(mg/L)**	55.12 ± 1.01	T0 (control)	49.62 ± 2.29c	39.29 ± 1.29d	41.23 ± 1.22d
		T1	51.86 ± 2.56b	44.00 ± 1.39bc	46.82 ± 1.56bc
		T2	52.0 ± 2.26a	44.62 ± 1.57ab	47.82 ± 1.41ab
		T3	51.55 ± 2.22b	43.08 ± 2.49c	45.93 ± 1.62c
		T4	52.38 ± 2.29a	45.19 ± 2.45a	48.33 ± 1.01a
**Total antioxidant activity (mmol Trolox equivalent/L)**	0.3 ± 0.001	T0 (control)	0.20 ± 0.001c	0.30 ± 0.01d	0.35 ± 0.01d
		T1	0.2.5 ± 0.02b	0.51 ± 0.21bc	0.48 ± 0.02bc
		T2	0.2.75 ± 0.016a	0.55 ± 0.01ab	0.59 ± 0.05ab
		T3	0.2.43 ± 0.03b	0.41 ± 0.02c	0.45 ± 0.04c
		T4	0.30 ± 0.005a	0.63 ± 0.01a	0.61 ± 0.02a

Values are mean ± SEM (*n* = 10)

One way anova was applied to check the overall behavior of the study parameter to elaborate the effect of treatments on selected parameter of rats at the termination of study. To evaluate the differences among the mean LSD test was applied. Values in same column within each parameter with different letters were significantly different from each other (*p* ≤ 0. 05)

Study I: Normal rats

Study II: Hypercholesterolemic rats

Study III: Hyperglycemic rats

T_0_: Control (without active ingredients) T_1_: Containing Coriander seeds

T_2_: Containing Black seeds T_3_: Containing Fenugreek seeds

T_4_: Containing Coriander seeds+ Black seeds+ Fenugreek seeds

In study I, cholesterol non-substantially decline by 2.01, 3.01, 1.48 and 3.45% was observed in groups rely on T_1_, T_2_, T_3_ and T_4_, respectively as compared to control. In contrary, a significant reduction in cholesterol was recorded in study II, highest by T_2 (_11.95%_)_ followed by T_4_ (10.45%), T_1_ (8.15%) and T_3_ (6.32%) as compared to control_._ Similar trend was observed in study III, cholesterol momentously reduced in experimental groups with 7.01, 10.01, 5.12 & 8.41% by T1, T2, T3 & T4, respectively as compared to control.

In study I, minimum HDL level was noticed in T_0_ (39.85 ± 1.25 mg/dL) that non-significantly improved in T_3_ (0.21%), T_1_ (0.32%) and T_4_ (0.58%) groups however, maximum level (0.99%) was noticed in T_2_. Nonetheless in study II, HDL enhanced significantly from 59.23 ± 2.51 mg/dL (T_0_) to 4.09,4.45, 3.99 and 4.19% in T_1_, T_2_, T_3_ and T_4_, respectively (Table 3). Likewise, in study III, minimum HDL value 38.77 ± 1.23 mg/dL was observed in T_0_ which significantly uplifted in T_1_ (3.01%), T2 (3.55%), T3 (2.94%) and T4 (3.22%).

The LDL level affected significantly in all the studies by the experimental treatments. However, BCS based experimental diet caused most potent effect followed by the combination of all spices (CS + BCS + FS). The recorded decline in LDL in study I by TI, T2, T3 & T4 was 3.5, 5.2, 2.71& 4.5%, respectively. It is also worth mentioning that these spices based treatments alone and in combination worked promisingly in hyperlipidaemic state as compared to normal and hyperglycemic situations. In this context, the pattern of effectiveness was same, T2 caused maximum decline followed by T4, T1 and T3 as 12.23, 10.54, 7.54 & 3.24%, respectively. Moreover, in study III the recorded significant decline in T1, T2, T3 & T4 was 10.01, 12.01, 7.51 & 11.01%, respectively as compared to T0 (46.53 ± 0.99 mg/dL). Similar trend was observed in triglycerides levels of studied animals, all the treatments exhibited the diminishing effect however effect was more pronounced in hyperlipidaemic state as compared to hyperglycemic and normal phases. Moreover, BCS showed highest potential in this regard (Table 3).

#### Glycaemic management perspective of the treatments

The abnormal glycaemic responses are among the more evident health discrepancies and the studied compounds were evaluated for their glycaemic management perspective through glucose and insulin assessment in experimental rats. In contrary to lipid profile, the combination of spices T4 (CS + BCS + FGS) imparted more promising effect instead of T2 (BCS). Likewise, maximum effect was observed in Study III followed by study II and Study I.

In study I, glucose non-substantial decreased by 2.01, 3.01, 1.48 and 3.45% in T_1_, T_2_, T_3_ and T_4_ groups, respectively as compared to control. Nevertheless, momentous decrease in glucose was recorded during Study II i.e. 7.01% in T_1_, 8.41% in T_2_, 5.12% in T_3_ and 10.01% in T_4_. Similarly, in study III, rat fed with diet CSP + BCSP + FSP (T_4_) diminished the glucose level by 10.59%, in respective trials. Whereas diet comprised of BCSP (T_2_), CSP (T_1_) and FSP (T_3_) resulted 9.01, 8.15 and 7.32% glucose reduction, respectively. In study II, the treatments T_4_, T_2_, T_1_ and T_3_ resulted as 3.1, 2.25, 1.21 and 1.02% uplift in insulin, correspondingly. Similarly, in study III, maximum elevation 4.25% was observed in T_4_ followed by 3.27 and 3.01% in T_2_ and T1while minimum 2.99% in T_3_, respectively (Table 3).

#### Effect of treatments on indicators of antioxidant status

For the estimation of antioxidant potential of tested compounds, the total antioxidant activity through TAC and glutathione assay were measured. It is worth mentioning that again combination of spices (T4) performed better as compared to their alone treatments.

In study I, the minimum glutathione level was noticed in T_0_ (49.62 ± 2.29 mg/L) that momentously elevated in T_1_ (4.53%), T_2_ (4.99%), T_3_ (3.89%) and T_4_ (5.58%). Likewise, in study II, the glutathione in T_4_ (15.02%) was substantially increased as compared to T_0_ (39.29 ± 1.29 mg/L). Whereas, the recorded enhancement in T1, T2 and T3 were 11.99, 13.59 & 9.66%, respectively. Similarly, in study III, glutathione values were uplifted from 41.23 ± 1.22 mg/L (T_0_) to 17.23% (T_4_), 15.99% (T_2_), 13.56% (T_1_) and 11.42% (T_2_), respectively (Table 3). Similar significant enhancement was observed in TAC level in all studies by the treatments and order of effectiveness was CS + BCS + FS > BCS > CS > FS. It is also well elaborated by the results that maximum uplift was noticed in hyperlipidemic rats followed by hyperglycemic and normal rats.

#### Liver function tests and haematological analysis

**Fig. 5:**
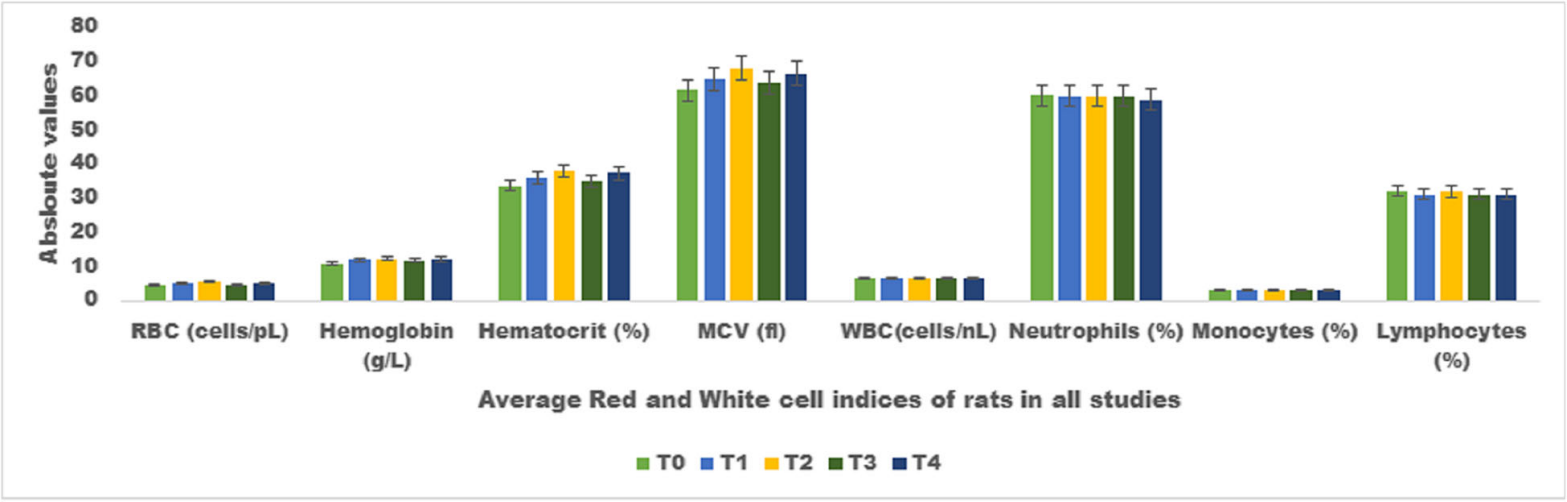
Effect of treatments on Absolute Average values for Hematological indices (Red blood cell indices and White blood cell indices) of all studies at the termination of trial (56th days). The experimental diets T0 Group rely on respective Experimental Diet without active ingredient), T1 (Group rely on respective Experimental Diet+ Coriander seed supplementation @ 1 g/Kg B.W) T2 (Group rely on respective Experimental Diet+ Black Cumin seed supplementation @ 1 g/Kg B.W), T3 (Group rely on respective Experimental Diet+ Fenugreek seed supplementation @ 1 g/Kg B.W) & T4 (Group rely on respective Experimental Diet+ Coriander Seed+ Black cumin seed + Fenugreek seed supplementation @ 0.333 + 0.333 + 0.333 g/Kg B.W) were given through entire study period. Values are mean ± SEM (*n* = 10) and level of significance were determined at (*p* ≤ 0. 05)

To understand the magnitude of lipid per oxidation and safety concerns liver functioning tests like ALT, AST and ALP were assessed. The high cholesterol and high fructose diets caused substantial abnormalities in the concentration of these enzymes owing to accelerate the lipid peroxidation. Nonetheless, the spices based intervention caused significant decline in the abnormal level of ALP, AST & ALT in all the studies owing to their strong antioxidant potential. The order of reactivity in all studied were CS + BCS + FS > BCS > CS > FS (Table 3). Moreover, it is also well observed from the recorded values that the supplementation of these compounds in the experimental diets did not cause any deleterious effect thus validating the safety of these bioactive moieties. This trend is further well supported by the outcomes of hematological analysis indicating the no abnormal effect of these to the Red and white blood cell indices (Fig. 5).

## Discussion

### In vitro characterization

In the recent exploration we might be first time investigated the synergistic role of Black cumin, coriander seed and fenugreek seed against lipidemic related abnormalities both in vitro and in vivo*.* The hallmark of the research is the extensive in vitro characterization, HPLC quantification and in vitro pancreatic lipase inhibitory effect estimation of tested compounds followed by a bioefficay trial. The outcomes of studies exhibited that all the spices elucidated strong antioxidant potential not in alone but also in their combination. The outcomes of project showed higher efficiency of methanol for antioxidant indices extraction as compared to water. Polyphenol estimation is the initial step to validate the antioxidant potential of the product. In this milieu, spices unveiled appreciable amount of phenolic contents which is well reflected through the findings of previous studies for instant, Ghosh et al. [29] investigated the TPC in fenugreek, black cumin and coriander seeds in methanol extract and noticed higher in black cumin then the rest. Likewise observation were reflected in the findings of Souri et al. [30], they were of the view that the polyphenol contents are varied as a function of method of extraction, solvent to material ratio and polarity of the solvents [31, 32]. The FRAP and ABTS are the most widely adapted assays to estimate the lipid peroxidation diminishing perspective of the tested compounds. In this study, the higher activity of combination of spices may be owing to the varied structural diversity, more Functional groups and multiple mode of mechanisms. The strong antioxidant activity of spices has been corelated through the presence of active ingredients. The major bioactive molecule in black cumin is thymoquinone while funergreek seed and cumin seed contain diosgenin & phenolic acids, respectively Khole [33] Iqbal & Alam [34, 35]. Dietary fat absorption is mainly dependent upon the activity of pancreatic lipase enzyme that accelerate the hydrolysis of triacylglycerol to 2-monoacylglycerol and fatty acids. It is manifest from the outcome that all the tested extracts caused momentous diminish in the activity of this enzyme showing the effectiveness of these against lipid related abnormalities.

### In vivo performance

In the present study we found the significant impact of spices based dietary intervention on abnormal lipid profile and glycaemic responses in normal, hypercholesterolemia and hyperglycaemic rats. However, more potent effect was noticed in hypercholesterolemia rats then the rest. It is also important to narrate that BCS based intervention showed more potent effect against lipid related abnormalities whereas combination of CS + BCS + FGS performed better in glycaemic and antioxidant capacity indicators. The polyphenol rich products usually caused reduction in feed and drink intake owing to their effect on appetite and satiety. The less feed consumption in rats relies on spices based dietary intervention is reported by the work of different scientists [36, 37]. The spices based dietary intervention has been proved effectual for weight reduction through multiple mechanistic routes. In this context, accelerate lipogenesis, promoting the rapid bile acid secretion, increase fat oxidation and enhancing the thermogenesis are among the notable once [1, 17, 38]. The current results regarding the reduced body weight in spices administrated rats are concordant with the earlier work of Soliman [39] reported reduction in weight of Sprague Dawley rats after provision of 500 mg/kg black cumin seeds. The most likely observed fact in the animal models (rats, rabbits etc.) treated with spices is the momentous reduction in the liver cholesterol and this is due to the maximum degradation of cholesterol to bile acids than its synthesis [40]. The results in present study concerning significant decrease in cholesterol are in harmony with the earlier work of Sultan [41] investigated the effect of black cumin seed powder as a hypoglycemic agent by utilizing @ 1 and 2% and noticed momentous reduction in cholesterol levels. It is inferred that decrease in cholesterol level is due to reduced synthesis of cholesterol by hepatocytes or by diminishing its fractional reabsorption from the small intestine. Likewise, Al-Saleh [42] deduces that black cumin seeds comprises substantial amount of sterols, especially β-sitosterol that has the ability to inhibit absorption of dietary cholesterol. Furthermore, PPARα (Peroxisome Proliferator-Activited Receptor) activation also concluded to minimize the cholesterol [4, 43, 44]. Likewise, enhancement in LCAT (plasma lecithin cholesterol acyl transferase) and HMG-CoA (hydroxyl methyl glutaryl CoA reductase) activity by spices caused improved degradation of cholesterol [45]. The optimum level of LDL is necessary to maintain a healthy human life. Black cumin seed based diet provision showed significant decline in the elevated LDL level in rats possibly due to presence of ter-butylhydroquinone and thymoquinone [46, 47]. Thymoquinoe (Active ingredient of *Nigella sativa* seeds) imparted significant impact on Doxorubicin, which induces hyperlipidemic nephropathy in rats, lowering triglycerides and total cholesterol [48].

Likewise, the spices reduce the LDL biosynthesis in liver by hindering the 3-hydroxy-3-methylglutaryl coenzyme activity [49]. Spices effectiveness against glycaemic abnormalities has been attributed to their suppressing effect on different carbohydrate metabolism enzymes like pyruvate kinase, lactate dehydrogenase, phosphofructokinase and hexokinas isozymes Type I, II and [50, 51]. Furthermore, presence of steroid saponin compounds and alkaloids possess insulin sensitizing effects inhibiting in vitro sodium-dependent intestinal glucose uptake [52]. The coriander seed consumption for a period of 60 days caused significant decline in elevated blood glucose level of human subjects. Provision of coriander seed powder @ 5 g/day twice for a period of 60 days exhibited 13% decline in glucose level. The spices imparted supressing impact on glucose level due to either inhibition of glucosidase and amylase activity in the gastrointestinal tract or the suppression of glucose absorption by preventing intestinal nutrient transporters like intestinal Na-dependent glucose transporter 1 (SGLT1). Likewise, black cumin seed consumption @ 300 mg/kg/day proved beneficial against insulin sensitivity in drug induced diabetic rats [53]. The black cumin consumption enhanced the insulin insensitivity and improving extra pancreatic actions of insulin, reducing the oxidative stress, preserve pancreatic β-cell integrity leading to improved insulin levels. Later, Arshad observed improvements in glycemic abnormalities and inferred that this perspective is ascribed the spices ability to reduce the leptin concentration and increase in adiponectin concentration. The fenugreek consumption improved the activity of glucose transporters (GLUTs); major carriers that maintain the glucose homeostasis and require IRβ and AMPKR proteins for their translocations further enhancing muscle, liver, and adipose cell glucose uptake [54, 55].

Antioxidant status of biological sample is mainly associated with the beneficial impact of the applied compound. In this study we observed significant enhancement in the antioxidant status revealed by increase glutathione and total antioxidant capacity (TAC) of animals rely on spices based intervention as compared to control. This veracity might be the possible mechanism by which spices divulged the modulating role against hyperlipidaemia and hypercholesterolemia related disparities because these situations are the outcomes of the oxidative stress thus provision of antioxidant rich products proved significant for their control. Our findings are in accordance with the previously reported effects of these spices on total antioxidant status of experimental rats [56, 57]. The statement is well supported by the findings of Inove [58] deduced that black cumin seeds uplift the GSH levels in the islet β cells protecting the membranes against oxidative damage by regulating the redox status of proteins in the membrane. In the current study we observed the significant decline in the elevated liver function enzymes and no deleterious effect on blood indices thus proved the safety of these spices based intervention. The hyperlipidaemia and hyperglycaemia related phases induced oxidation in body that effect the liver, structural and functional integrity caused elevation in liver enzymes concentration in the serum. Being a strong source antioxidant, spices imparted reduction in this elevation by scavenging the free radicals and removal of oxidation promotors.

## Conclusions

It is inferred from the outcomes that spices showed promising compositional and nutritional profile both in alone and combination. Among the different solvents, methanol was more effectual than the rest. All the spices exhibited good antioxidant profile. Thymoquinone an active ingredient in BCS and disogenin in FS was found, whereas chlorogenic acid, caffeic acid and kaempferol were maximum in CS. They exhibited a dose dependent inhibition in pancreatic lipase activity indicating their in vitro suitability against fat related abnormalities. The bio-evaluation study on Sprague Dawley rats concluded that BCS impart excellent lipidemic response whereas CS + BCS + FS showed effective glycaemic effect. However, it is suggested as future recommendation that the outcomes of *in vitro* antioxidant activity may discourage owing to their less biological value. Moreover, in vivo human clinical trial ought to be conducted to access the actual therapeutic impact of these spices. Likewise, for the estimation of glutathione, and lipid profile indicators, more sophisticated methods should be adapted**.** Moreover, it is also strongly suggested that the in vivo studies should be conducted to understand the mechanistic targets associated with hypoglycaemic and hypolipidemic perspectives of spices.

## Data Availability

The dataset supporting the conclusions of this article is included within the article.

## Abbreviations

ALP: Alkaline phosphatase
ALT: Alanine transaminase
AMPK: Activation of activated protein kinase
AST: Aspartate aminotransferase
GLTU: Glucose transporters
GPX: Glutathione peroxidase
GSH-Px: Glutathione peroxidase
GST: Glutathione-S-transferase
HDL: High density Lipoprotein
IRβ: Insulin receptor β subunit
LDL: Low density Lipoprotein
MDA: Malondialdehyde
OHdG: 8-hydroxy-2′ -deoxyguanosine
ROS: Reactive oxygen spices
BC: Black Cumin
FG: Fenugreek
CS: Cumin seed
TG: Triglycerides
VLDL: Very low density Lipoprotein
